# Publisher Correction: Stateful characterization of resistive switching TiO_2_ with electron beam induced currents

**DOI:** 10.1038/s41467-018-02844-6

**Published:** 2018-01-24

**Authors:** Brian D. Hoskins, Gina C. Adam, Evgheni Strelcov, Nikolai Zhitenev, Andrei Kolmakov, Dmitri B. Strukov, Jabez J. McClelland

**Affiliations:** 1 Center for Nanoscale Science and Technology, National Institute of Standards and Technology, Gaithersburg, MD 20899 USA; 2 Materials Department, University of California Santa Barbara, Santa Barbara, CA 93106 USA; 3 Electrical and Computer Engineering Department, University of California Santa Barbara, Santa Barbara, CA 93106 USA; 4 Institute for Research and Development in Microtechnologies, 077190 Bucharest, Romania; 5 Institute for Research in Electronics and Applied Physics, University of Maryland, College Park, MD 20742 USA

Correction to: *Nature Communications* 10.1038/s41467-017-02116-9; Article published online: 07 December 2017

The original version of this Article contained an error in Eq. 1. The arrows between the symbols “T” and “B”, and “B” and “T”, were written “↔” but should have been “→”, and incorrectly read:

$$I_{{\mathrm{EBIC}}} = I_{{\mathrm{EBAC}}} + I_{{\mathrm{SEE}}} + I_{\left( {{\mathrm{e}} \leftrightarrow {\mathrm{h}}} \right)} + I_{{\mathrm{ISEE}}}^{{\mathrm{T}} \leftrightarrow {\mathrm{B}}} + I_{{\mathrm{ISEE}}}^{{\mathrm{B}} \leftrightarrow {\mathrm{T}}}$$

The correct from of the Eq. 1 is as follows:

$$I_{{\mathrm{EBIC}}} = I_{{\mathrm{EBAC}}} + I_{{\mathrm{SEE}}} + I_{\left( {{\mathrm{e}} \leftrightarrow {\mathrm{h}}} \right)} + I_{{\mathrm{ISEE}}}^{{\mathrm{T}} \to {\mathrm{B}}} + I_{{\mathrm{ISEE}}}^{{\mathrm{B}} \to {\mathrm{T}}}$$

This has now been corrected in both the PDF and HTML versions of the article.

