# Supplementary material for: Stateful characterization of resistive switching TiO2 with electron beam induced currents
Source: Nat Commun. 2017 Dec 7;8:1972. doi: 10.1038/s41467-017-02116-9 (PMC5719452; doi:10.1038/s41467-017-02116-9)
Supplement: Supplementary file 1 — Supplementary Information [file 41467_2017_2116_MOESM1_ESM.pdf]

## Supplementary Information

## Supplementary Note 1: More Fabrication Details

To minimize topographic effects, eliminate charging in the scanning electron microscope, and maximize the signal, a device structure was developed involving a thin device weakly electrically coupled to the silicon wafer through a thin dielectric. The devices are a true parallel plate capacitor (Supplementary Figure 1), as opposed to the typically used crosspoints, and electrical contact is made from above.

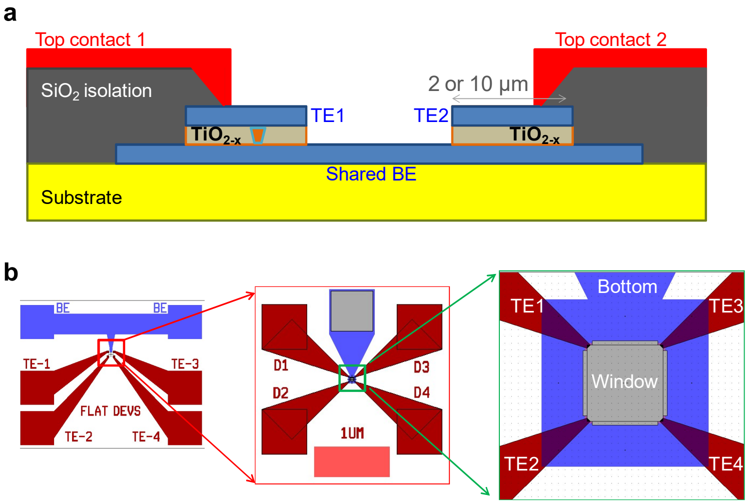


**Supplementary Figure 1.** Desired structure (a) and optimized mask design showing big contacts connected to small contacts that are connected to 4 devices.

For ease of processing, the bottom electrode is patterned into a plate on the surface floating on a thin dielectric of $Al_2O_3$ (3 nm) and $TiO_2$ (2 nm). Deposited above this layer is a dielectric active region. Above this layer, patterned into an approximately 1.7 µm × 1.7 µm square, is a top electrode. Above this layer is 100 nm of dielectric passivation, but with two open windows for the bottom electrode contact as well as an open window which exposes the device region except covered with approximately 250 nm of overlay from the dielectric. Electrode contacts are deposited onto the top electrode with approximately 200 nm of further overlap. The device top electrode is subsequently almost entirely revealed except for the contact and dielectric overlays. Another layer of macroscale contacts extend the electrodes further out into electrodes so the devices can be easily contacted by hand. To maximize wafer yield, each individual die contains 4 devices on a common bottom electrode.

The active layer was deposited in either a standard or inverted configuration with either a Ti or TiN getter layer. Prior to deposition, the bottom electrode was always oxygen plasma cleaned for 3 minutes *in-situ.*

Both top and bottom electrode are deposited by blanket sputter deposition. A 100 mm 525 µm thick Si wafer was used for processing. The bottom electrode is deposited onto a reactively sputtered $Al_2O_3/TiO_2$ blanket adhesion layer (3 nm/2 nm). The top electrode is deposited *in situ* after deposition of the active layer. Prior to patterning, the bottom and top electrodes are covered in a thin 20 nm layer of e-beam deposited $Al_2O_3$. Bottom and top

electrodes are patterned using a deep ultra-violet (DUV) lithography system (stepper with a 248 nm laser). A developable antireflective coating (spin speed 2500 rpm, bake 185 ºC, thickness approximately 50 nm) and a positive photoresist (spin speed 2500 rpm, bake 135 °C, thickness approximately 300 nm) were used for patterning. After exposure, the sample was held for an extended development whereby the TMAH present in the developer etches the $Al_2O_3$ at a rate of approximately 8 nm/min. After hard mask etching, the photoresist is removed in $O_2$ plasma and the devices are milled in an Ar ion mill system (IBE/RIBE/CAIBE system) to remove the metal. The final minute of etching is done in a mixture of Ar and $O_2$ to eliminate the effects of surface sputter reduction caused by the Ar on the $TiO_2$. The $O_2$ flow rate was selected over a binary search of post etch conductivity to determine the optimum flow rate of $O_2$ to eliminate bombardment induced conductivity in $TiO_2$. After patterning of the top electrode, the $Al_2O_3$ was left on rather than stripped to act as a protectant layer.

The dielectric passivation layers were patterned by DUV lithography, deposited in an e-beam evaporation system from $TiO_2$ and $SiO_2$ sources and lifted off (Supplementary Figure 3). The dielectric layer was composed of $TiO_2/SiO_2/TiO_2$ 10 nm/80 nm/10 nm. $TiO_2$ and $SiO_2$ were chosen for their good adhesion and low dielectric constant respectively. Preliminary tests showed that dry etching the passivation layer resulted in damage to the underlying device. Also as opposed to dry etching, it was found that e-beam evaporated layers produced, in the presence of photoresist undercut, a gentle slope ideal for deposition of top contact to the device top electrode. Sidewall deposition onto the photoresist appeared to be too mechanically unstable to sustain rabbit-ear formation.

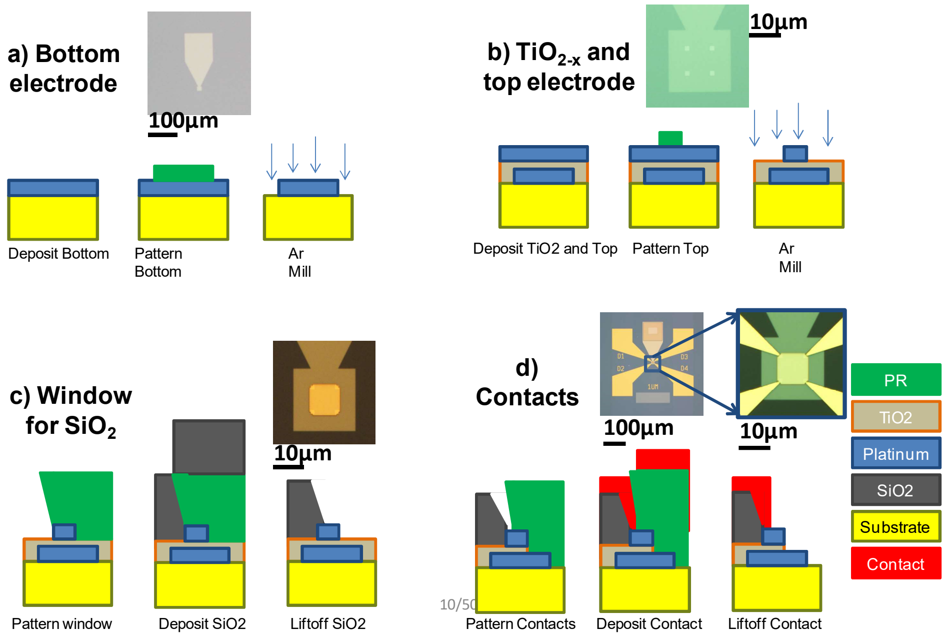


**Supplementary Figure 2.** a) Ar milling of the bottom electrode. b) Growth, patterning, and Ar milling of the top electrode. c) Patterning and e-beam deposition of the $SiO_2$ isolation. d) Patterning of the contacts to the top electrode.

The approximately 200 nm tall photoresist and approximately 100 nm dielectric layer produce a nearly 1:1 aspect ratio hole into which the top contact to the electrode must be deposited. The ideal gentle slope of the dielectric made normal angle deposition the choice for

electrode deposition, allowing filling of the contact area and continuous coverage from the top electrode onto the dielectric. 20 nm Pt electrodes were chosen so as to avoid contamination of the device due to diffusion of reactive species. A further 80 nm of Au buffering the Pt contact was selected due to its low mechanical stress and to ensure complete coverage of the dielectric slope. The extended macroscopic contacts were composed of e-beam Ti/Au (20 nm/1500 nm) patterned and lifted-off with contact lithography and a transparency mask. The final steps included wafer dicing and storage in anti-static boxes. For safe transport, devices were vacuum sealed in an $N_2$ ambient.

## Supplementary Note 2: More Measurement Setup Details and Image Processing

The microscope used for the measurement is an SEM with a $ZrO_2$/W Schottky emitter. Custom flanges and electrical connections connect the device to an external current amplifier and an external source measurement unit (SMU). Custom connections attach to a Faraday cup mounted to the stage and a custom sample holder. PTFE sample screws mount copper clips which simultaneously hold and electrically connect a sample. The sample was elevated by an epoxy mounted glass slide. The entire stage is electrically floating with respect to ground as is. To ensure a good common ground, the microscope and computer DAC were connected to the current amplifier chassis. The source measurement unit was left floating relative to the amplifier due to a large ground loop otherwise present, which generated a large 60 Hz noise signal.

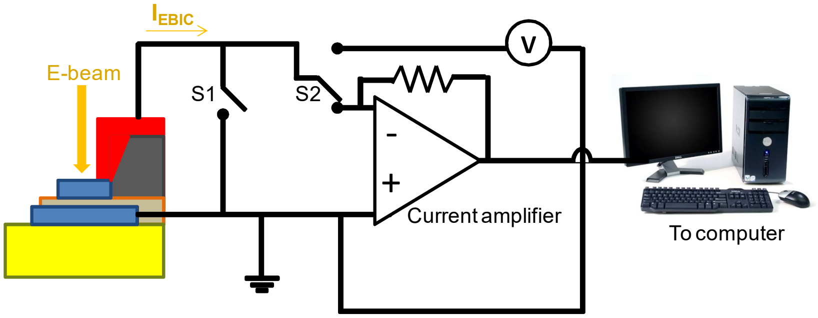


**Supplementary Figure 3.** Schematics describing EBIC measurement system and its principles during imaging. S1 is a grounding connection to short the device prior to adjusting the measurement setup. S2 switches between the current amplifier and an SMU voltage source for programming.

The measurement box (Supplementary Figure S4) consisted of a series of switches which included a switch to ground everything prior to switching between connections, a 5 kΩ series resistor used during forming (not shown), a switch to access the Faraday cup (not shown), and finally an interconnect switch go access the SMU or the current amplifier. Current amplifier data acquisition was done through a software system.

The optimum current amplifier settings were experimentally chosen to ensure the lowest possible input impedance and highest frequency. Typical settings include 200 nA $V^{-1}$ or 20 nA $V^{-1}$ signal amplification with a bandwidth of 20 kHz. 520 × 512 pixel images were acquired for an approximately 50 μs integration time per pixel. Current amplifier settings from 1 μa $V^{-1}$ to 20 nA $V^{-1}$ showed approximately the same value for generated current in the low resistance state.

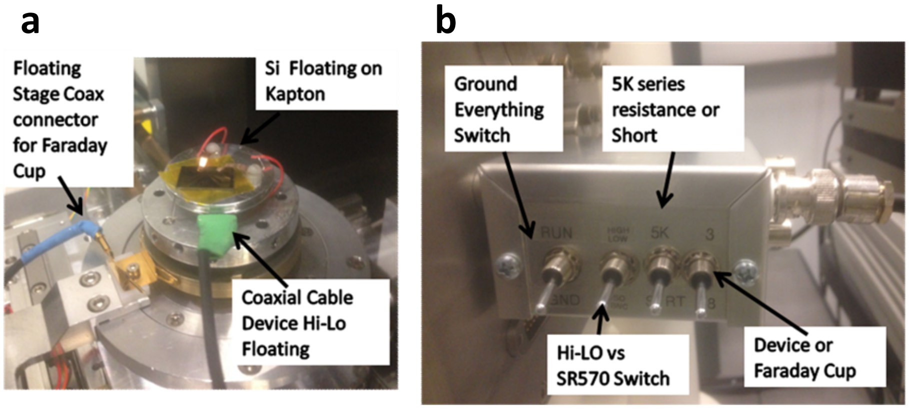


**Supplementary Figure 4.** Sample mounting (a) and switch box (b) used for the EBIC system at NIST

Low vapor pressure carbonaceous species are a significant problem in electron microscopy from the perspective of sample and chamber contamination. Early experiments showed severe carbon contamination which led to poor image quality, particularly at low keV. To make low keV image acquisition possible, an in-situ plasma cleaning system was installed on the microscope. The chamber and sample was verified to be cleaned using the NIST standard imaging test of imaging for 10 minutes at 200,000x and verifying that no carbon deposition was visible (Supplementary Figure 6)[1].

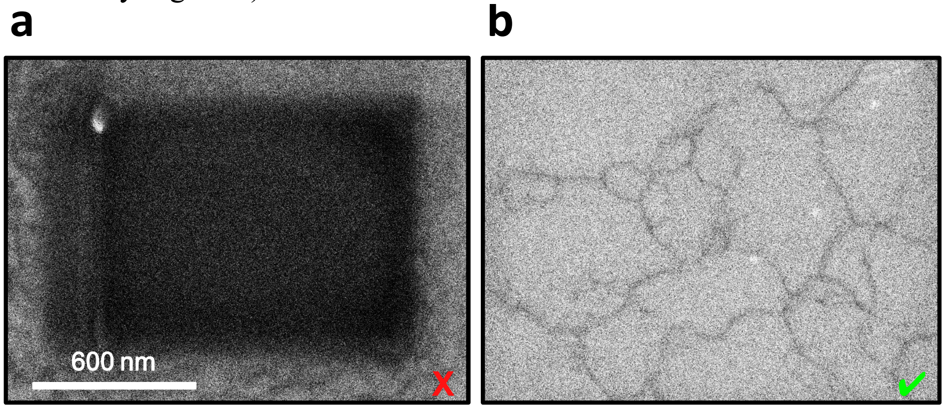


**Supplementary Figure 5.** Image stability conditions (a) with no plasma cleaning and (b) with plasma cleaning.

An important element of EBIC data acquisition is data recovery from a noisy environment. Since the measurements are conducted in the standard DC configuration without a lock-in, digital signal processing is necessary to remove artifacts present in the measurement. The various artifacts include 60 Hz noise, device noise, and read disturbances due to beam-sample interaction.

60 Hz noise is introduced by the EBIC system since the commercial acquisition system doesn't have any kind of buffered input, making noise from the acquisition computer system unavoidable. Software settings are selected to synchronize data acquisition with this noise source leading to well defined vectors for the noise in k-space. Taking the Fourier transform of the image yields an image with well defined noise peaks. A mask was implemented which zeros out the dominant sources of noise as well as scan artifacts along the $k_x = 0$ and $k_y = 0$ directions.

Read disturbances are due to small changes in the device state from the beam-sample interaction. Since the input impedance to the current amplifier is nonzero, any small change in the device state will induce a slight shift in the measured quantity, generating an offset. Also, instability in the amplifier can from time to time result in an offset in the zero value, and it was recorded to change from time to time. To eliminate this effect, mean line leveling was implemented under the assumption that the areas around the filament are at a constant current signal. Any artificially induced offset can be eliminated by adding an offset to all lines that should have a constant mean.

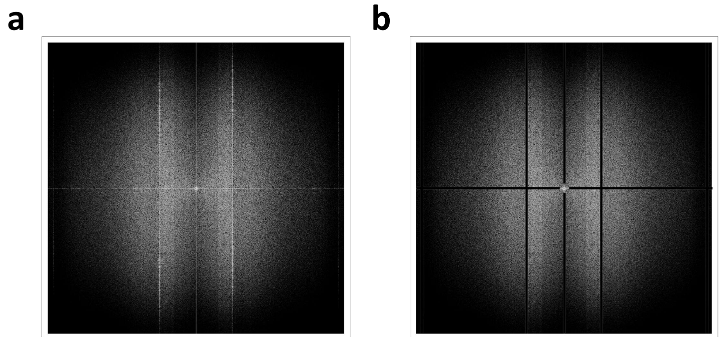


**Supplementary Figure 6.** Fourier masking to zero out the dominant sources of noise. (a) unmasked Fourier transform of the image; (b) masked Fourier transform of the image. The images have been enhanced for clarity.

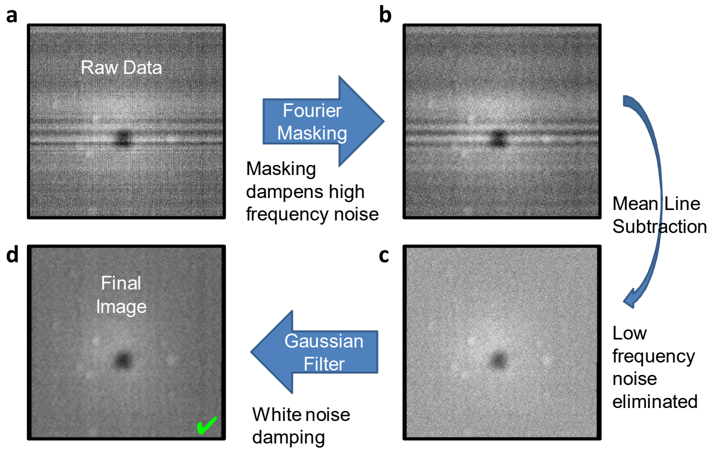


**Supplementary Figure 7.** Image processing algorithm to recover data. (a) An example of raw data. (b) An image after Fourier masking of the raw data. (c) elimination of low frequency noise or errors by subtracting the mean variation from approximately constant regions. (d) Gaussian blurring of background noise.

An important element of the data acquisition is alignment of a large number of acquired images during stateful data acquisition. Since the EBIC images change, it's necessary to use the companion SEM images to align the data. Algorithmically, the images are all stored in a data-structure with the offset information built in. Least squares minimization is used to align the images over a search of the various offset vectors. The offset vectors are then stored and the images are aligned. A small program was also used to align images in the event the algorithm failed, though this only occurred ~5% of the time.

## Supplementary Note 3: More Monte Carlo Simulation Details

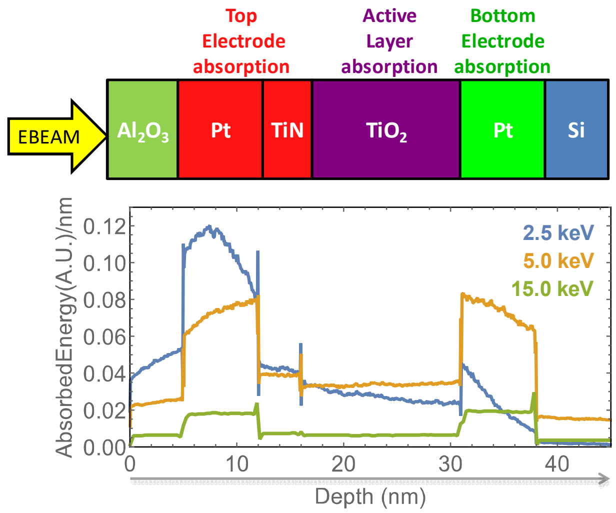


**Supplementary Figure 8.** Layer by layer description of the model with modeled absorption/nm as a function of beam energy and depth. Much greater absorption occurs in the denser Pt layers, and, at lower beam energy, more absorption occurs in the bottom than does in the top layers. At higher beam energies the experiment becomes effectively transmissive.

In the Monte Carlo Simulations, *Casino* was used to simulate $10^4$ electrons with energies from 0.25 keV to 25 keV (0.25 keV/step) were simulated for the two different structures, the standard and inverted. Typical atomic densities for the modeled compounds (Supplementary Table 1) were used with an energy cutoff of 0.05 keV. The energy absorbed at 0 keV was defined as 0. After extracting the data, the data was interpolated and then smoothed.

| **Supplementary Table 1: Densities of Materials** | | | | | |
|---|---|---|---|---|---|
| **Material** | **$Al_2O_3$** | **Pt** | **TiN** | **$TiO_2$** | **Ti** |
| **Density ($g/cm^3$)** | **3.6**[2] | **21.45**[3] | **5.6**[4] | **3.82**[5] | **4.5**[3] |

In Supplementary Figure 9, the absorbed energy for each layer is given a sign determined by the expected direction of the generated current as a function of absorption of energy in that layer. The top and the bottom electrodes both inject electrons into the dielectric, whereas the hole-pair EBIC signal in $TiO_2$ is determined by the work function difference between the two electrodes. Experimental EBIC data also show a similar peak energy absorption for the dielectric layer. Since a non-zero internal secondary electron emission current was detected even at the lowest beam energies, the residual protected $Al_2O_3$ layer is included in the total energy integration for the top electrode.

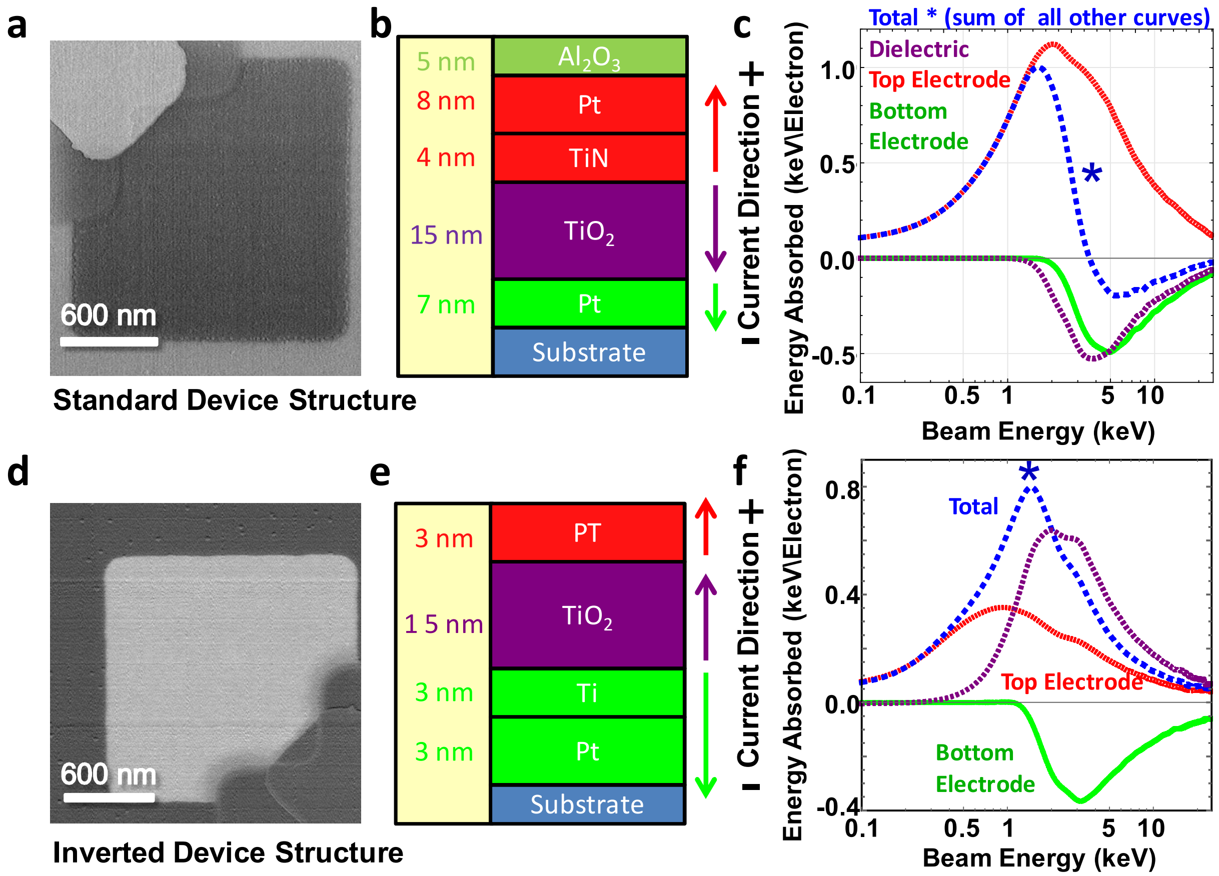


**Supplementary Figure 9.** (a) Electron beam induced current micrograph of a standard asymmetric device in the off state at 5keV incident energy. Dark contrast indicates negative current flow. (b) Cartoon schematic of the device structure in (a) with arrows indicating expected direction of current flow for energy absorption in each part of the device color coded by red (Top Electrode), purple ($TiO_2$ layer), and green (bottom electrode). (c) Energy absorption curves as a function of energy for each layer. (d) Electron beam induced current micrograph of a standard asymmetric device in the off state at 2.5keV incident energy. Bright contrast indicates positive current flow. (e) Cartoon schematic of the device structure in (a) with arrows indicating expected direction of current flow for energy absorption in each part of the device color coded by red (Top Electrode), purple ($TiO_2$ layer), and green (bottom electrode). (f) Energy absorption curves as a function of energy for each layer.

## Supplementary Note 4: Symmetric device Background Commentary

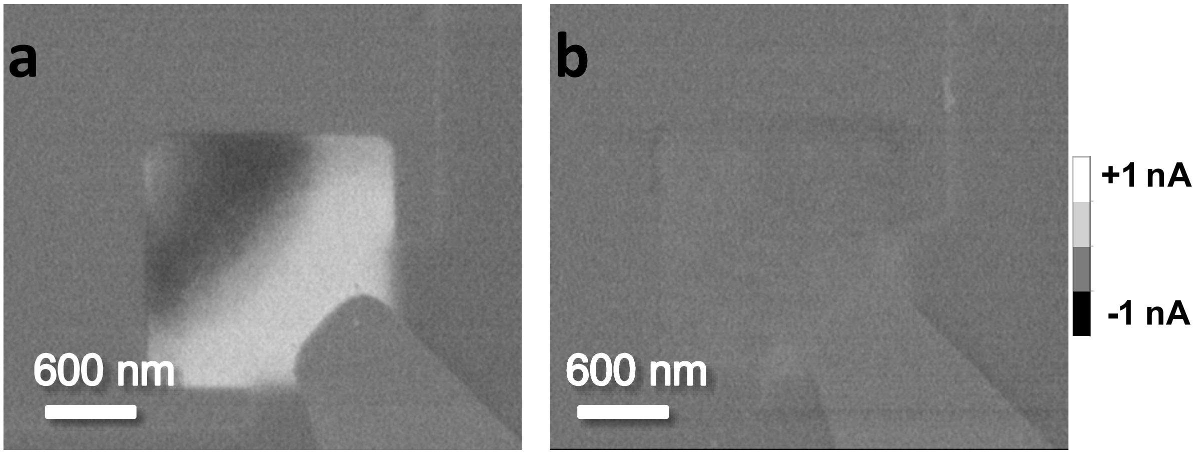


**Supplementary Figure 10.** (a) A virgin symmetric device soon after being exposed to an electron beam and (b) a virgin device after a few minutes of dosing with the electron beam

The symmetric parallel plate devices had an identical structure to the standard devices except without the TiN or Ti getter layer. The most interesting feature of these devices was the variations in background EBIC signal observed both across devices and within devices. Some devices exhibited background signal similar to the standard or inverted device, but many of them also appeared to exhibit instability in the current generation. Supplementary Figure 10 shows the EBIC signal of a device before and after extensive imaging. Initially the device exhibits a substantial signal, but it has huge gradations across the device not observed in the asymmetric structures. These gradations appeared to wave during video acquisition and then fade away resulting in Supplementary Figure 10.B. This kind of instability was not observed in the other device structures.

The origin of the relaxation is not clear, but it's possibly due to relaxation of defects excited by the beam. The resultant EBIC signal, after the sample has fully relaxed is substantially reduced as compared to the initial configuration.

## Supplementary Note 5: More Forming Details

While some devices could be formed without extensive device damage, poor yield was a severe problem during the device testing. This is well known to be due to parasitic capacitance and can only be mitigated by using an appropriate RF measurement setup or by integrating the device with a transistor[6]. Since the peak power during forming can be estimated as:

$$P_{\mathrm{peak}} = \sigma_{\mathrm{on}} V_{\mathrm{form}}^2 \qquad \text{(Supplementary Equation 1)}$$

Where $P_{\mathrm{peak}}$ is the peak power, $\sigma_{\mathrm{on}}$ is the on-state conductance after forming, and $V_{\mathrm{form}}$ is the forming voltage, reducing the forming voltage can lead to quadratic reductions in the total dissipated power during the forming process.

It was found that simultaneous application of voltage and the incident e-beam could lead to local increases in the conductivity of the device. To maximize this effect, the aperture was opened to allow an approximately 500 pA beam current at 5 keV (near the energy absorption maximum for the standard device) while voltage was applied. Supplementary Figure 11.a details a shift in the device conductivity with the simultaneous application of beam and voltage. This effect was exploited in Figure S12.b-f to reduce the device damage during forming. To form the devices, a current-controlled loop was initiated and limited by a 5 kΩ series resistor while a 5 keV beam was irradiating a region of the device.

Supplementary Figure 11.b and Supplementary Figure 11.c show extensive damage and bubble formation as a consequence of forming without the beam. Supplementary Figure 11.d and Supplementary Figure 11.e show dramatic reduction of the damage limited to ripening of the device by applying the beam during a 30 $\mu A\ s^{-1}$ ramp. S12.f and S12.g show even further reduction of the damage after applying a 1 $\mu A\ s^{-1}$ ramp (not shown) to 20 $\mu A\ s^{-1}$ followed by another 30 $\mu A^{-1}$ ramp leading to device forming. The damage was limited to a slight tearing of the upper corner. In addition to reducing the forming voltage, it was also possible to choose the filament location by placing the beam at a desirable location, typically in the center of the device. For the device in Figure 5 of the paper, a voltage ramp procedure similar to a previously published algorithm but without a series resistor[7]. The pulse width was 100 ns, helping to mitigate the device overshoot.Further research will clarify the mechanism of the forming voltage reduction, but it's likely related to defect generation or to a reduction in the formation energy of defects[8]

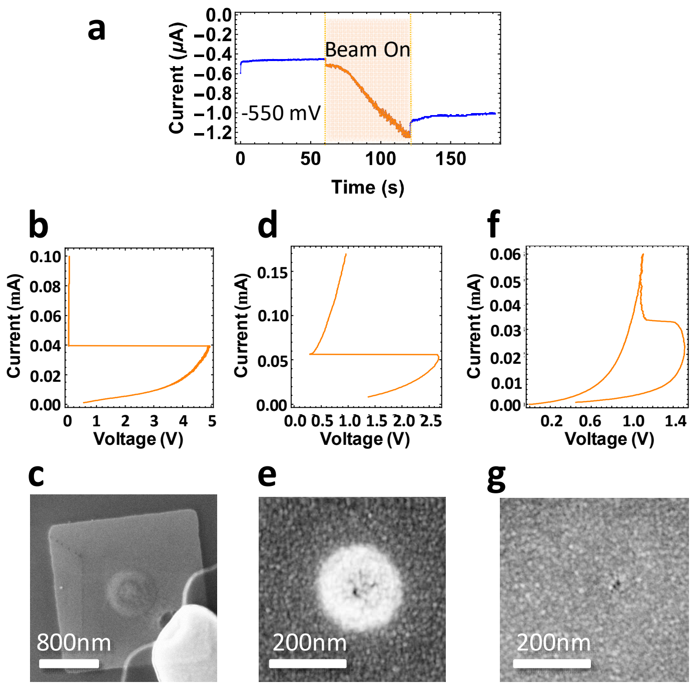


**Supplementary Figure 11.** (a) Effect of simultaneous beam exposure and applied voltage on the device. (b) A device formed in the absence of the beam. (c) Post forming damage of device formed in (b). (d) A device formed rapidly in the presence of the beam and (e) associated device damage. (f) A device formed slowly in the presence of the beam with (g) minimal damage. The 5 kΩ series resistor was mathematically removed from the measured data.

## Supplementary Note 6: More Spectral Characterization

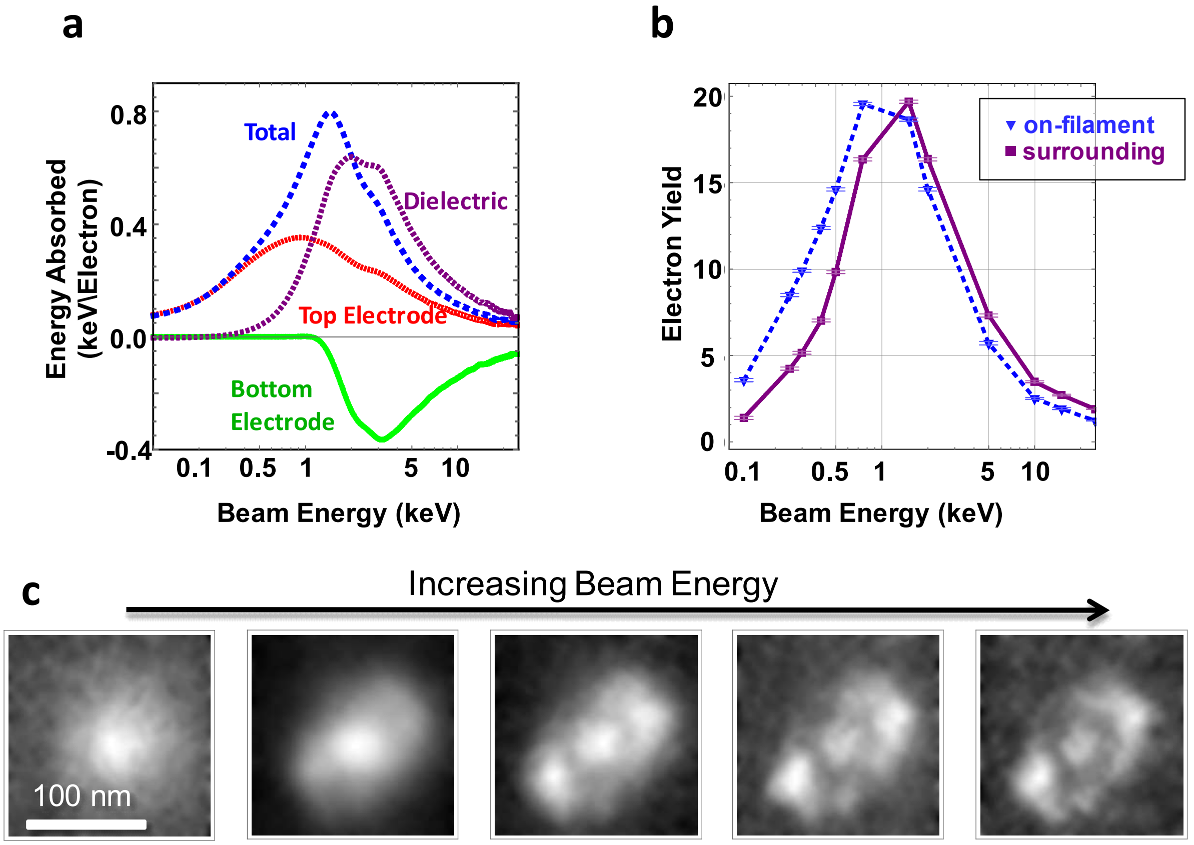


**Supplementary Figure 12.** Energy dependent excitation in an inverted device structure. This figure shows that there is only one polarity in this device for the filament signal and background. (a) Monte Carlo simulations of absorption in different parts of the structure as a function of energy. (b) Measured Electron yield as a function of energy. Error bars are standard deviation of the mean within a 11 × 11 pixel area at two different device locations near the filament and the periphery. Energy maximum is shifted to higher energy off the filament region (c) Energy dependence of EBIC images of the switching region.

Supplementary Figure 12 depicts the energy dependent current generation in an inverted device structure. Due to the absence of dark contrast, there is no way to categorically distinguish the filament current generation from the film generation. Doing energy dependent measurements shows that the central region appears to have significantly higher contrast at lower energies, implying it is the filament region, as would be predicted from internal secondary electron emission. These devices were not heavily studied since, in addition to the inverted structure, the top electrodes were also very thin (approximately 3 nm Pt) and subject to the mechanical and thermal in stability. As a result, switching was very difficult without damaging the top electrode.

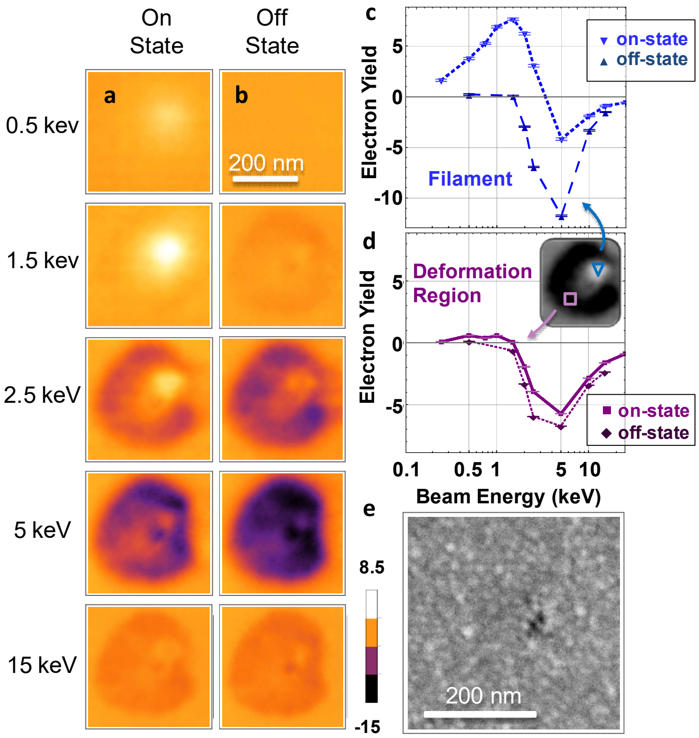


**Supplementary Figure 13.** Energy dependent excitation in a standard device structure. Note this device was used in the Figure 9 of the paper. (a) EBIC yield micrographs of a device in the on-state. Filament is clearly visible by high ISEE signal at low beam energy (b) EBIC micrographs of a device on the off-state with surrounding recrystallized region visible at 5 keV energy. (c) Measured electron yield as a function of energy on the filament region. (d) Measured electron yield as a function of energy off the filament region. Error bars are standard deviation of the mean within a 11 × 11 area at two different device locations near the filament and the periphery. (e) Micrograph of imaged region showing three small holes near the filament.

Supplementary Figure 13 depicts the energy dependent current generation of the device from Figure 9 of the main paper. Due to a different device history than the device from Figure 5, there are a few differences in the magnitudes of the different currents. However, all of the features are the same, including the approximate energies of signal maximum and minimum.

### Supplementary Note 7: More Spectral Characterization

Tabular presentation of important images used in the paper. The switching process is arrayed top to bottom. Each EBIC Image is taken after each associated switching curve.

**Supplementary Figure 14**. Off state transition data:

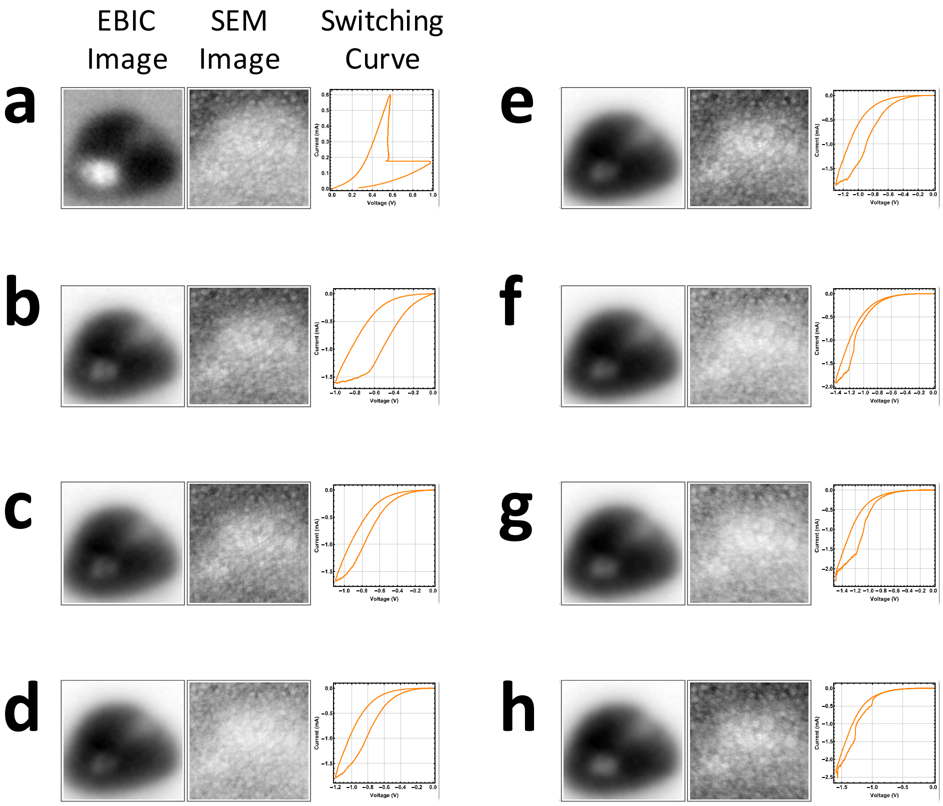

**Supplementary Figure 15**. On state transition data:

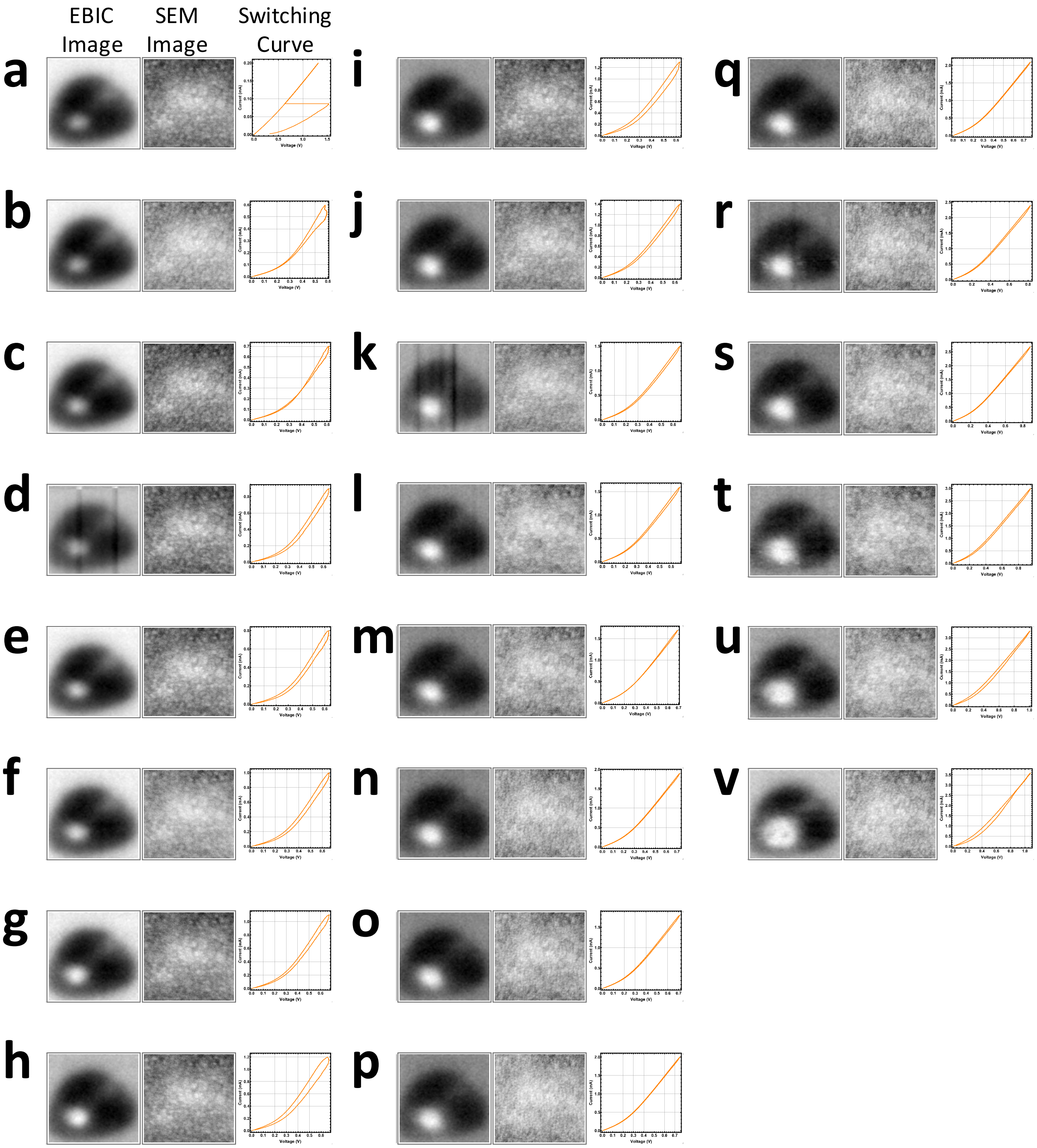

**Supplementary Figure 16**. Reversible polarity data

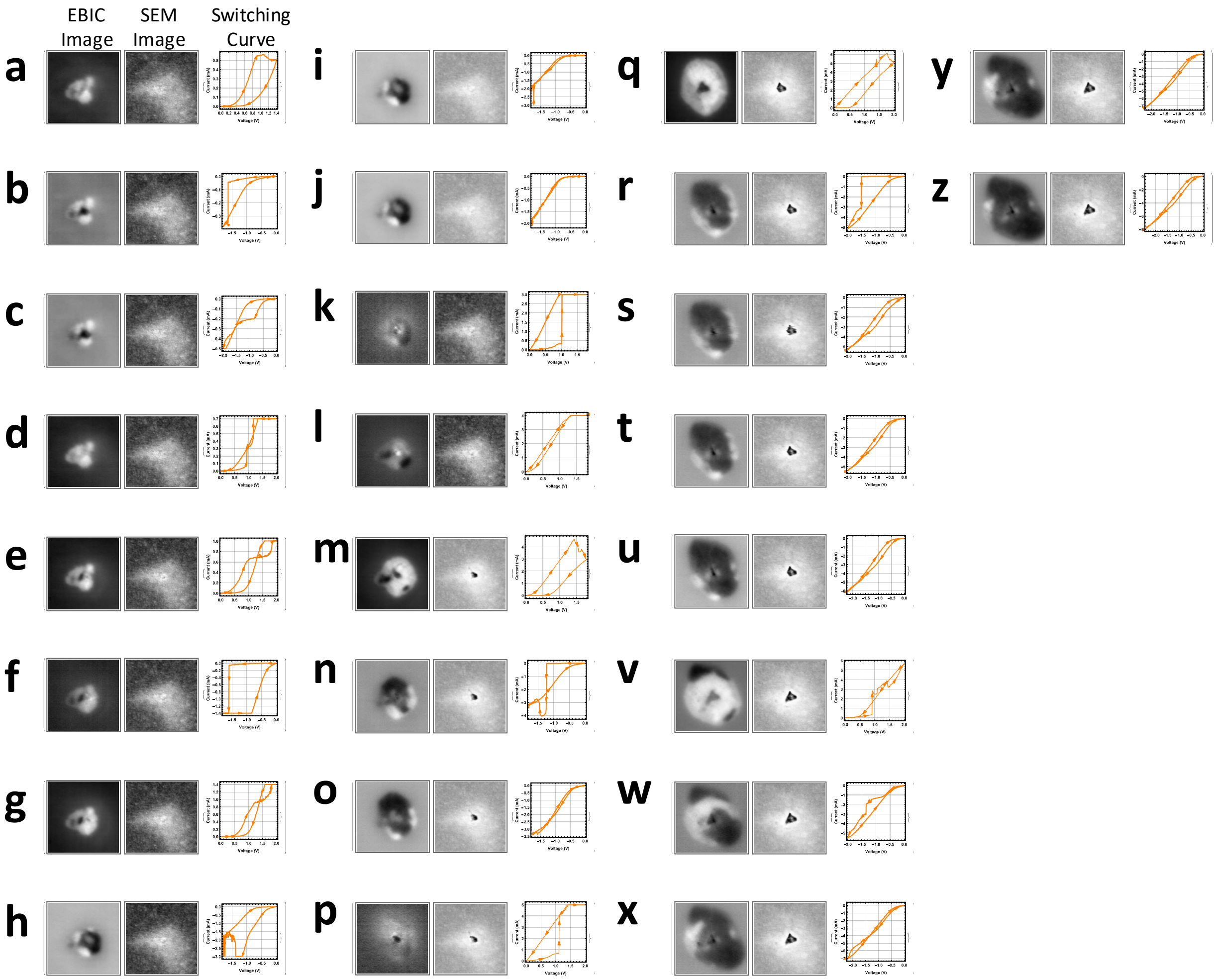

## Supplementary References


1. Vladar, A. & Postek, M. Electron Beam-Induced Sample Contamination in the SEM. *Microsc. Microanal.* **11** (2005).
2. Koski, K., Hölsä, J. & Juliet, P. Properties of aluminium oxide thin films deposited by reactive magnetron sputtering. *Thin Solid Films* **339**, 240-248 (1999).
3. Callister, W.D. & Rethwisch, D.G. Fundamentals of Materials Science and Engineering: An Integrated Approach, 4th Edition: An Integrated Approach. (Wiley, 2011).
4. Patsalas, P., Charitidis, C., Logothetidis, S., Dimitriadis, C.A. & Valassiades, O. Combined electrical and mechanical properties of titanium nitride thin films as metallization materials. *J. Appl. Phys.* **86**, 5296-5298 (1999).
5. Anderson, O., Ottermann, C.R., Kuschnereit, R., Hess, P. & Bange, K. Density and Young's modulus of thin TiO2 films. *Fresenius' Journal of Analytical Chemistry* **358**, 315-318 (1997).
6. Yi Meng, L. et al. Elimination of high transient currents and electrode damage during electroformation of $TiO_2$ -based resistive switching devices. *J. Phys. D: Appl. Phys.* **45**, 395101 (2012).
7. Fabien, A., Ligang, G., Brian, D.H. & Dmitri, B.S. High precision tuning of state for memristive devices by adaptable variation-tolerant algorithm. *Nanot* **23**, 075201 (2012).
8. Egerton, R., Li, P. & Malac, M. Radiation damage in the TEM and SEM. *Micron* **35**, 399-409 (2004).
