# Supplementary material for: Stateful characterization of resistive switching TiO2 with electron beam induced currents
Source: Nat Commun. 2017 Dec 7;8:1972. doi: 10.1038/s41467-017-02116-9 (PMC5719452; doi:10.1038/s41467-017-02116-9)
Supplement: Supplementary file 3 — Description of Additional Supplementary Files [file 41467_2017_2116_MOESM3_ESM.docx]

**Description of Additional Supplementary Files**

File Name: Supplementary Movie 1

Description: Figure 8 switching: A compilation of electron beam induced current micrographs summarizing the switching in Figure 8.
