# Supplementary figures and images for: Stateful characterization of resistive switching TiO2 with electron beam induced currents
Source: Nat Commun. 2017 Dec 7;8:1972. doi: 10.1038/s41467-017-02116-9 (PMC5719452; doi:10.1038/s41467-017-02116-9)

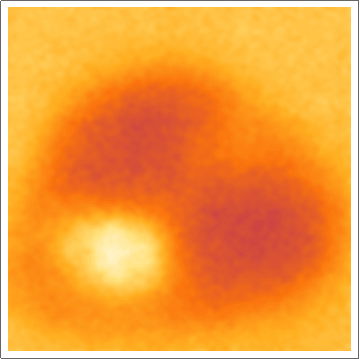

Supplement: Supplementary file 4 — Supplementary Movie 1 [file 41467_2017_2116_MOESM4_ESM.gif]
